# Efficacy and Safety of Inhaled Ethanol in Early-Stage SARS-CoV-2 Infection in Older Adults: A Phase II Randomized Clinical Trial

**DOI:** 10.3390/pharmaceutics15020667

**Published:** 2023-02-16

**Authors:** Ana Castro-Balado, Ignacio Novo-Veleiro, Néstor Vázquez-Agra, Gema Barbeito-Castiñeiras, Ana Estany-Gestal, Rocío Trastoy-Pena, Miguel González-Barcia, Irene Zarra-Ferro, María Carmen del Río-Garma, Carlos Crespo-Diz, Olga Delgado-Sánchez, Francisco J. Otero-Espinar, Cristina Mondelo-García, Antonio Pose-Reino, Anxo Fernández-Ferreiro

**Affiliations:** 1 Pharmacy Department, University Clinical Hospital of Santiago de Compostela (SERGAS), 15706 Santiago de Compostela, Spain; 2 Clinical Pharmacology Group, Health Research Institute of Santiago de Compostela (IDIS), 15706 Santiago de Compostela, Spain; 3 Pharmacology, Pharmacy and Pharmaceutical Technology Department, Faculty of Pharmacy, University of Santiago de Compostela (USC), 15782 Santiago de Compostela, Spain; 4 Internal Medicine Department, University Clinical Hospital of Santiago de Compostela (SERGAS), 15706 Santiago de Compostela, Spain; 5 Microbiology Department, University Clinical Hospital of Santiago de Compostela (SERGAS), 15706 Santiago de Compostela, Spain; 6 Epidemiology and Clinical Research Unit, Health Research Institute of Santiago de Compostela (IDIS), 15706 Santiago de Compostela, Spain; 7 Clinical Analytic Department, University Clinical Hospital of Santiago de Compostela (SERGAS), 15706 Santiago de Compostela, Spain; 8 Pharmacy Department, University Clinical Hospital of Pontevedra (SERGAS), 36162 Pontevedra, Spain; 9 Sociedad Española de Farmacia Hospitalaria (SEFH), 28001 Madrid, Spain

**Keywords:** inhaled ethanol, elderly, COVID-19, SARS-CoV-2, randomized controlled trial

## Abstract

Background: Inhaled ethanol in the early stages of SARS-CoV-2 infection may reduce the viral load, decreasing progression and improving prognosis. The ALCOVID-19 trial was designed to study the efficacy and safety of inhaled ethanol in older adults at initial phases of infection. Methods: Randomized, triple-blind, placebo-controlled phase II clinical trial. Experimental group (n = 38) inhaled 65° ethanol through an oxygen flow, while in the control group (n = 37), water for injection was used. General endpoint was to evaluate disease progression according to the modified World Health Organization (WHO) Clinical Progression Scale. Specific effectiveness endpoints were body temperature, oxygen saturation, viral load assessed by cycle threshold (Ct) on real-time polymerase chain reaction (RT-PCR), analytical biomarkers and use of antibiotics or corticosteroids. Specific safety outcomes were the absence of ethanol in plasma, electrographic, analytical, or respiratory alterations. Results: In the intention-to-treat population, no differences were found regarding disease progression. Mean Ct values increased over time in both groups, being numerically higher in the ethanol group, reaching a value above 33 only in the ethanol group on day 14, a value above which patients are considered non-infective. No differences were found in the other specific effectiveness endpoints. Inhaled ethanol was proven to be safe as no plasma ethanol was detected, and there were no electrocardiographic, analytical, or respiratory alterations. Conclusions: The efficacy of inhaled ethanol in terms of the progression of SARS-CoV-2 infection was not demonstrated in the present trial. However, it is positioned as a safe treatment for elderly patients with early-stage COVID-19.

## 1. Introduction

In the early stages of the COVID-19 pandemic outbreak, rapid spread and high mortality forced the scientific community to research against the clock [1]. Serious efforts were made worldwide to rapidly develop effective vaccines, which have been responsible for the change in the paradigm of the disease [2]. Prior to vaccination, the elderly population was especially susceptible to SARS-CoV-2 infection, in which severity and number of deaths have had a greater impact [3,4]. The percentage of hospitalizations in the first wave was high, and over 95% of total deaths occurred in people older than 60 years, with more than 50% of all deaths being people aged 80 years or older [5,6].

As a consequence of this dramatic situation, clinicians have been forced to resort to alternatives such as compounded formulations and drug repositioning. Numerous studies have been carried out in humans focused on previously approved drugs for the treatment of other pathologies [7,8], including antiviral agents, monoclonal antibodies and inflammation inhibitors/antirheumatic drugs, among others [9]. However, only a small number of drugs have been approved by drug agencies for the treatment of COVID-19.

There is a large amount of evidence demonstrating the antiviral effects of ethanol with a graduation between 62–71° [10,11], possibly due to solvent action on lipids and protein denaturation [12,13,14,15,16]. In early stages of the disease, viral replication occurs in the epithelial layer of the upper respiratory tract [17]. The use of inhaled ethanol could act as an antiseptic in the upper airways against SARS-CoV-2 at initial stages, reducing viral load more rapidly than in the natural course of infection with no systemic adverse effects [15]. Since inhaled ethanol is not commercialized, its preparation as a compounded formulation and galenic characterization was previously carried out by our group [18].

The current phase II clinical trial received the approval from the Spanish Agency of Medicines and Medical Devices to test the efficacy-safety of the 65° ethanol formulation in elderly patients with COVID-19 (ALCOVID-19; EudraCT number: 2020-001760-29) [19].

## 2. Materials and Methods

### 2.1. Trial Design

A randomized, triple-blind, placebo-controlled phase II clinical trial was conducted in a geriatric ward in Spain between 26 October 2020 and 19 March 2021 (when the last patient completed follow-up). The trial was approved by the institutional review boards of the participating institutions and the autonomous region of Galicia (registration number: 2020/218), being supervised by an independent data and safety monitoring board.

### 2.2. Trial Patients

Starting in September 2020, a geriatric institution was transformed into a low-complexity inpatient unit for mildly ill residents infected with SARS-CoV-2 from other nursing homes. Patients aged 65 years or older who tested positive by real-time polymerase chain reaction (RT-PCR) test for SARS-CoV-2 at the time of screening were identified, and their eligibility was assessed.

Patients had to be in the initial stage of the disease, with <7 days of symptoms, absence of dyspnea and pneumonia, oxygen saturation (SpO_2_) in room air >93% or partial pressure of oxygen >70 mmHg and respiratory rate <25 per minute. Exclusion criteria included severe respiratory disease, chronic broncho-pneumopathy, or potential diseases that may interfere with inhaled experimental treatment. The complete list of inclusion and exclusion criteria is detailed in Table S1 of the Supplementary Material. All participants agreed to participate by signing the informed consent.

### 2.3. Randomization and Intervention

The experimental treatment of 65° hydroalcoholic ethanol (ethanol 65% (*v*/*v*)) compounded formulation or water for injection (control) were elaborated at 20 °C and packaged in humidifiers in the hospital pharmacy department, under sterile conditions, as described in our previous work [18]. Preparations were assigned to the corresponding batch to maintain the blind and sent to the geriatric ward. Allocation of treatments was carried out randomly in a 1:1 ratio according to their order of inclusion on a master randomization list.

Patients in the experimental group received oxygen at 2 L/min moistened with the 65° ethanol compounded formulation contained in the humidifier (INTL CE0482. Ref. 3230, Oximesa Nippon Gases, Madrid, Spain) connected to a Ventimask^®^ (Flexicare Medical Ltd. Mountain Ash, UK), every 8 h (inhalation time 15 min) for 5 days. In the placebo group, water for injection was used instead. In both groups, recommended treatment according to the protocol of COVID-19 clinical management established by the Spanish Ministry of Health at the beginning of the trial was administered concomitantly.

### 2.4. Data Collection

**Figure 1:**
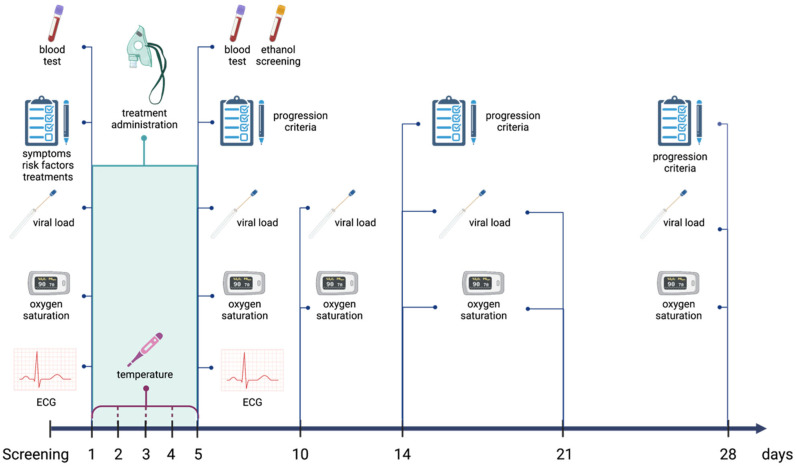
Trial design: study visits, collected variables and administration of the study medication. ECG: electrocardiogram. Created with BioRender.com.

Study visits were scheduled from the day-1, or baseline visit, when the subject received the study medication or placebo for the first time. The duration of participation was 28 days divided into 8 visits on days 1, 2, 3, 5, 10, 14, 21 and 28. Data were recorded into the web application Research Electronic Data Capture (REDCap) (Vanderbilt University, Nashville, TN, USA), as shown schematically in Figure 1. On day 1, data related to risk factors, symptoms and chronic treatments were collected. Disease progression was assessed according to the modified World Health Organization (WHO) Clinical Progression Scale (Table S2) on days 5, 14 and 28, as well as hospital admissions, and duration of hospitalization. SpO_2_ was registered on days 1, 5, 10, 14, 21 and 28, while temperature and antipyretic treatment were registered once daily on each day of experimental treatment administration.

Nasopharyngeal samples were taken with a swab and virus transport medium (UTM^®^, COPAN Diagnostics Inc., Murrieta, CA, USA) on days 1, 5, 10, 14, 21 and 28. RNA extraction (Hamilton STARlet, Reno, NV, USA) and RT-PCR (Allplex 2019 nCoV Assay, Seegene, Republic of Korea) detecting the genes E, RdRP and N were performed. The RT-PCR provides a cycle threshold (Ct) value for each of the genes, a semi-quantitative measure which represents how many PCR cycles are required to detect RNA [20], with higher values corresponding to low viral load and vice versa. The present technique has a specificity of 100% and a limit of detection of 100 RNA copies/PCR reactions [21]. A Ct value of 40 is undetectable and considered the lower limit of detection. For the analysis, the arithmetic means of Ct values between the three genes in each patient were calculated. Ct values of the N gene were also analyzed separately, as this is the most conserved one [22]. A patient who achieves a Ct value above 33 after a previous positive test was considered non-infectious for SARS-CoV-2 [23,24], allowing the isolation to be lifted.

Blood tests were performed at the baseline visit and at the end-of-treatment visit (day 5), including basic biochemical parameters, complete blood count, coagulation study, and inflammation/infection biomarkers. These parameters were classified as indicators of treatment efficacy and/or safety, as shown in Table S3 [25,26]. It should be noted that some of the safety biomarkers have also been described as prognostic indicators of the disease [25,26]. Concomitant treatments were recorded during the study. For the analysis, corticosteroid doses were equated to equivalent doses of prednisone [27]. On the end-of-treatment visit, blood ethanol was determined. An electrocardiogram was performed on days 1 and 5. Occurrence of adverse effects was recorded during the entire study period.

### 2.5. Trial End Points

#### 2.5.1. General Outcome

The general outcome was to evaluate the progression to stages of greater severity of COVID-19 according to the modified WHO Clinical Progression Scale (Table S2) [28].

#### 2.5.2. Specific Effectiveness Outcomes

One of the prespecified specific outcomes related to treatment efficacy was to evaluate the evolution of hypoxemia using the SpO_2_ with pulse oximetry. Normalization of body temperature, defined as axillary temperature <37.5 °C without antipyretic treatment, was measured once daily during treatment administration days.

Evolution of viral load was estimated and evaluated indirectly through Ct of the SARS-CoV-2 genes, E, RdRP and N. Biomarkers associated with COVID-19 disease progression were studied to assess the efficacy of the experimental treatment at the baseline visit and at the end-of-treatment visit (day 5) (Table S3). Lastly, the need for antibiotics and corticosteroids were also considered during the entire study period.

#### 2.5.3. Specific Safety Outcomes

Blood ethanol determination was carried out on the last day of treatment to verify the absence of systemic absorption. To ensure cardiovascular safety, an electrocardiogram was performed during baseline and end-of-treatment visit (day 5) and, to assess the systemic safety, liver and renal function were monitored at baseline and end of treatment (Table S3). Information regarding the appearance of any adverse effect was collected during the administration of the experimental treatment, as well as in the subsequent follow-up visits.

### 2.6. Early Trial Termination

To contain SARS-CoV-2 infections among institutionalized elderly patients when the number of COVID-19 cases was high, a specific center was opened to house all the positives detected in nursing homes for elderly people and avoid hospital overcrowding. However, this center was closed when the number of cases decreased after vaccination, forcing the early termination of the clinical trial.

### 2.7. Statistical Analysis

Hospitalization rate in COVID-19 patients over 60 years was about 70%. With this intervention, it was considered that it could decrease to 40%. A superiority limit of 10% was set, so that it would be necessary to include 71 patients/group to ensure a power of 80% to draw conclusions with a significance level of 5%. Considering expected losses of 10%, it would be necessary to include 78 patients/arm, 156 in total. Statistical analysis was performed with SAS^®^ 9.4 (SAS Institute Inc., Cary, NC, USA). Normality was studied using the Shapiro–Wilk test. Quantitative variables with normal and non-normal distributions were compared using independent *t*-test and Mann–Whitney test, respectively; variables with normal and abnormal distributions for paired groups were compared using paired *t*-test and a Wilcoxon signed-rank test, respectively. Qualitative variables were compared using Chi-square or Fisher’s exact test. Statistically significant values are considered those whose *p*-value is <0.05.

## 3. Results

### 3.1. Trial Population

**Figure 2:**
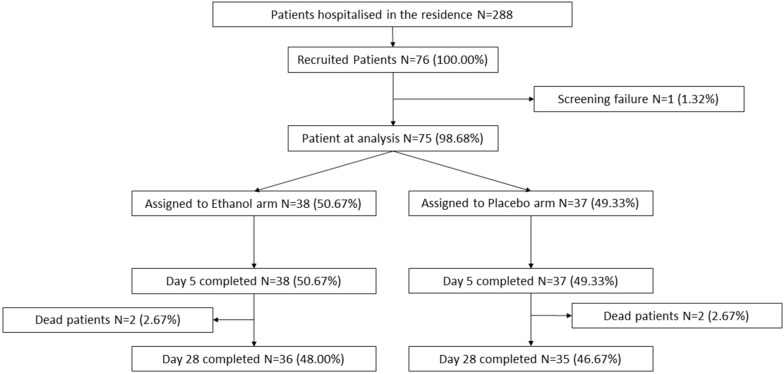
Trial flow chart showing patient enrollment, group allocation and deaths.

From 26 October 2020 to 19 March 2021, a total of 76 patients met the eligibility criteria and provided informed consent. One patient became ineligible before enrollment. Therefore, 75 patients with SARS-CoV-2 infection underwent randomization and were included in the intention-to-treat analysis; 38 were assigned to receive inhaled 65° ethanol and 37 inhaled placebo (Figure 2).

The median age was 83.0 years (IQR: 77.0–89.0), with 62 patients (82.7%) older than 75 years. A total of 51 patients (68.0%) were women. Baseline demographic characteristics and risk factors are summarized in Table 1. The most prevalent chronic pathology was hypertension (69.3%), followed by diabetes mellitus (26.3%) and cardiovascular disease (24.0%). Only half of the patients included presented symptoms (Table 1), with the most frequent being general discomfort (34.7%) and cough (36.0%).

Chronic treatments at the baseline visit, classified by therapeutic groups according to the Anatomical Therapeutic Chemical Classification System (ATC), are shown in Table S4. The most frequent were antithrombotic agents (86.7%) and drugs against peptic ulcer and gastroesophageal reflux (92.0%). Differences between groups were found in antidepressants, prescribed to a greater extent in the placebo group (*p* = 0.0133), and in drugs used in benign prostatic hypertrophy, with more prescriptions in the ethanol group (*p* = 0.0253).

### 3.2. General Outcome

The number of patients per group classified according to the modified WHO Clinical Progression Scale at days 1, 5 (end of treatment), 14, and 28 are shown in Table S5. Most patients were classified in severity stages ranging from ambulatory mild disease to hospitalized with moderate disease. Notably, no patient progressed to the more severe stages of hospitalization requiring high-flow oxygen therapy or mechanical/invasive ventilation techniques in either of the two groups.

**Figure 3:**
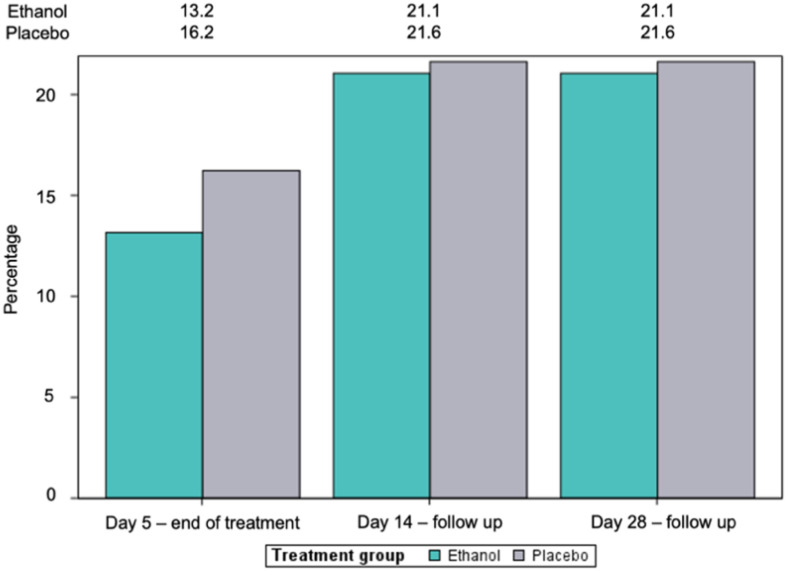
Cumulative relative frequency (%) of patients in both treatment groups who progress according to the modified WHO Clinical Progression Scale. Percentages were calculated based on the populations of each group (n = 38 in ethanol group and n = 37 in placebo group).

Results are shown graphically in Figure 3, where the cumulative percentage of progressions with respect to the previous visit in both treatment groups is represented. Statistical analysis showed no differences in disease progression between groups (Table S6), indicating that progression to more severe stages of the disease occur in a similar way regardless of treatment received.

A total of 7 patients (19.4%) in the ethanol group and 6 (16.7%) in the placebo group required hospital admission. The median time from study inclusion to hospitalization was 6 days (IQR: 3.0–9.0) and 6.5 days (IQR: 4.0–7.0), respectively. The average days of hospitalization were 10.5 (SD: 7.1) in the ethanol group and 8.5 (SD: 8.5) in the placebo group, although this difference was not significant. Two patients died in each group, in the ethanol group before day 14, while in the placebo group they died between days 14 and 28.

### 3.3. Specific Effectiveness Outcomes

#### 3.3.1. Oxygen Saturation, Temperature, and Antipyretic Treatment Administration

The evolution of SpO_2_ and the presence of hypoxia are shown in Tables S7 and S8, with median values ranging between 95–96% in both groups. Regarding body temperature, median values did not exceed 37 °C at any study visit (Table S9). The administration of antipyretic treatment is shown in Table S10. No statistically significant differences were found in any of these parameters.

#### 3.3.2. Evolution of Viral Load

**Figure 4:**
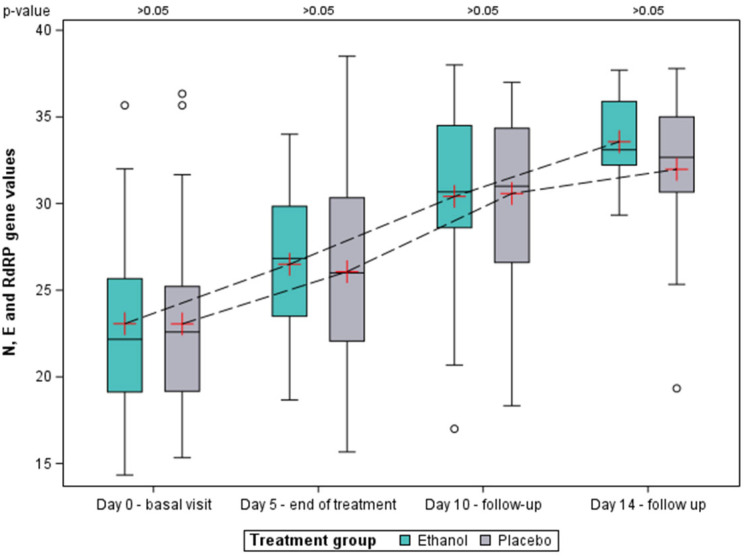
Cycle threshold (Ct) values of the three genes (N, E and RdRP) up to visit on day 14. Mean is represented by a red cross, median is represented by the horizontal line contained in the box, and the first and third quartiles by the lower and upper limits of the box, respectively. The mean Ct values at each visit are linked together by a dashed line for each of the groups.

Ct mean values of the three genes (N, E and RdRP) per patient was calculated and, subsequently, the mean for each of group at the baseline visit, and days 5, 10 and 14 was obtained (Figure 4). Mean Ct values for both groups were 23.1 at the baseline (SD ethanol: 5.0; SD placebo: 5.2), showing similar viral load. At subsequent visits, Ct values increased as viral load decreased, with ethanol group being nominally higher on days 5 and 14, and lower on day 10, compared to the placebo group, although differences were not statistically significant. The number of RT-PCR tests decreased considerably over the course of the study visits, as patients who had previously tested negative were not retested again. Due to this loss of data, no Ct values are reported for days 21 and 28.

Mean Ct values of the three genes on the day-14 visit were 33.6 (95% CI: 31.6–35.6) for the ethanol group and 32.0 (95% CI: 29.5–34.5) for the placebo group. The cut-off point of 33 Ct, which marks non-infectivity for SARS-CoV-2 after a previous positive test, was only exceeded by the ethanol group. Although these differences were not statistically significant, this would have an impact in terms of lifting isolation, facilitating the discharge of patients to their original nursing homes.

**Figure 5:**
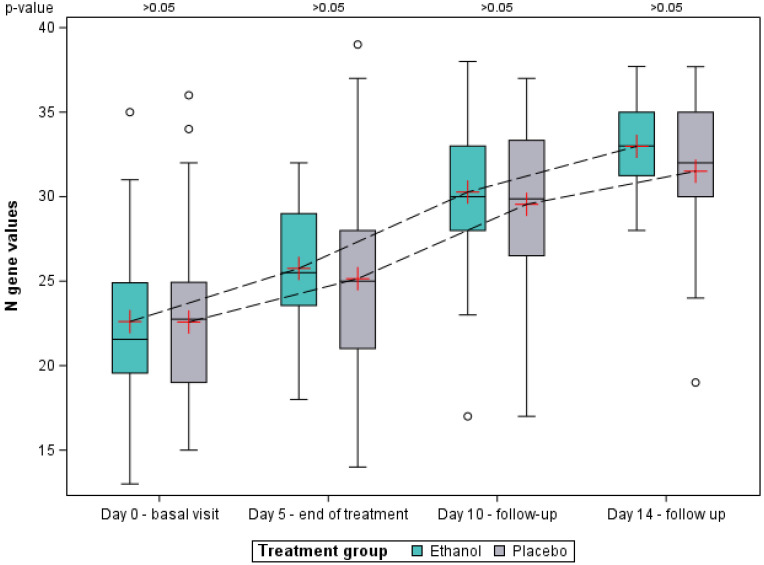
Cycle threshold (Ct) values oh the N gene up to visit on day 14. Mean is represented by a red cross, median is represented by the horizontal line contained in the box, and the first and third quartiles by the lower and upper limits of the box, respectively. The mean Ct values at each visit are linked together by a dashed line for each of the groups.

Similar behavior was observed with the N gene (Figure 5). In this case, no differences were found between groups at the baseline visit either, while the mean Ct values in the ethanol group were always numerically higher with respect to the placebo group for the study visits on days 5, 10 and 14. At day 14, the mean Ct value of the N gene in the ethanol group was 33 (95% CI: 30.8–35.2), while for the placebo group it was 31.5 (95% CI: 28.9–34.1). Despite this, statistical significance was also not reached for any of the study visits.

#### 3.3.3. Biomarkers Associated with COVID-19 Disease Progression

In terms of efficacy biomarkers, differences were only found in procalcitonin at baseline visit with higher values in the ethanol group (0.0306), while differences were not significant at the end-of-treatment visit (Table S11). Considering percentage of change between visits (Table S12), creatinine kinase was significantly reduced by 36.08% in the ethanol group versus 11.65% in the placebo group (*p* = 0.0020). Regarding C-reactive protein (CRP), it remained unchanged in the ethanol group, while it was increased in the placebo group by 50% (*p* = 0.0379). This difference was studied in greater depth, noting that in the ethanol group CRP was reduced in 48.6% of the patients, while in the placebo group it was reduced only in 28.6% of the participants, although the loss of data (n = 35 in both groups) hindered obtaining statistical significance.

#### 3.3.4. Use of Corticosteroids and Antibiotics

Corticosteroids were prescribed in 19 patients in each group (ethanol 50.0%; placebo 51.4%). The median time in days from study inclusion to initiation was 1.0 (IQR: 0.0–0.4) in the ethanol group and 2.0 (IQR: 0.0–0.4) in the placebo group, without statistical differences. Once started, the mean duration of treatment was 19.7 (SD: 13.4) and 15.5 days (SD: 11.6), respectively, also without statistical significance. Equivalent doses of prednisone/day and the total equivalent doses of prednisone are shown in Table S13.

Antibiotics were prescribed in 15 (39.5%) and 9 (24.3%) patients in the ethanol and placebo group, respectively, during the study period. The time from inclusion to the start of treatment was 4.6 (SD: 3.0) days on average in the ethanol group, and 5.2 (SD: 3.5) days in the placebo group. Antimicrobials most frequently prescribed during the study were beta-lactams, 18.6% among the total number of participants. Differences between groups were not statistically significant.

### 3.4. Specific Safety Outcomes

#### 3.4.1. Determination of Systemic Absorption of Ethanol

Administration of inhaled ethanol was not associated with the presence of ethanol at a systemic level, as all patients tested negative for ethanol in blood.

#### 3.4.2. Electrocardiogram and Ethanol Toxicity Biomarkers

None of the subjects presented electrocardiogram abnormalities at baseline or after 5 days of treatment. Moreover, in terms of liver- or renal-function markers, no differences were found between groups at baseline and day 5 visits (Table S11) nor in the variation between visits (Table S12).

#### 3.4.3. Occurrence of Adverse Effects

Neither during the administration of the experimental treatment nor in the subsequent follow-up period were adverse effects observed at the level of the respiratory system.

## 4. Discussion

This randomized, triple-blind, placebo-controlled phase II clinical trial sought to assess the efficacy and safety of inhaled 65° ethanol to treat early stages SARS-CoV-2 infection in elderly patients.

In view of the risk of mass infection in nursing homes in the early stages of the pandemic, when a patient tested positive, a screening RT-PCR was performed on the rest of the residents, this was the reason why only half of the patients had symptoms at the beginning of the study. Regarding the general objective, both groups uniformly progress to more severe stages. Moreover, it is worth mentioning that numerous cases of “negative symptoms” were detected in both groups, despite not showing clear symptoms of COVID-19 progression. After more than two years of pandemic, it has already been described how elderly patients can appear “off” with SARS-CoV-2 infection, with symptoms such as fatigue, weakness, lethargy, delirium, loss of appetite, apathy, confusion, agitation or disorientation [29,30]. The modified WHO Clinical Progression Scale used in this clinical trial does not allow the appearance of these symptoms to be associated with clinical deterioration in the elderly population, which is one of the main limitations detected in the present study. When patients showed this negative symptomatology, clinicians proceeded to prescribe corticosteroids, which resulted in clinical improvement. Therefore, those prescriptions could have been used as an indirect measure of the appearance of negative symptoms, but the lack of differences obtained between groups did not allow us to draw conclusive results.

As for viral load, in both groups there was an increase in Ct values as the study progressed related to the decrease in viral load. Considering the three genes together, for most of the study visits Ct values were higher in the ethanol group, although statistical significance was not achieved. This trend was also observed considering only the N gene alone, and seems to suggest that our treatment hypothesis was fulfilled, in which ethanol may be exerting its antiseptic effect in the upper airways [17]. Even so, this reduction in viral load did not translate into a lower incidence of disease progression, as previously discussed. These results should also be interpreted with caution, since the loss of data was greater in the later study visits. However, it is important to note that with a Ct value above 33, the patient is considered not infectious for SARS-CoV-2 [23,31], a value that was only reached in the ethanol group at visit 14, both taking into account the three genes or the N gene alone. This would imply that the ethanol group would reach a non-infectious condition earlier than the placebo group. Patients with a Ct value above this value may receive an early discharge as preventive isolation is lifted to avoid new infections, thus favoring the return of patients to their nursing homes of origin without risk of infecting other residents. If we were to transfer this to the general population, it could have much more relevant implications, associated with public health, sick leaves, reduction in isolation days, as well as cost savings. One of the limitations of the study is that viral viability was not known, as nasopharyngeal samples were not inoculated in cell culture. At the time of the clinical trial, all material and personnel resources were devoted to diagnostics, but viral culture is a very slow and complex process which cannot be used as a tool for clinical practice because it does not allow decision making. Therefore, transmissibility was monitored through Ct measurement by real-time PCR, from which infectivity can be inferred [32], which is the most sensitive method and endorsed by the Spanish Ministry of Health [24].

Regarding temperature, it should be noted that many patients had been treated with paracetamol or non-steroidal anti-inflammatory drugs (NSAIDs) prior to inclusion and during study as analgesics. This should be considered another limiting factor when it comes to detecting an association between fever and the use of antipyretics, since fever might have been masked.

In terms of the safety of the experimental treatment, it is very important to note that no adverse effects were observed during the administration or in the following weeks. Moreover, ethanol was not detected in the blood of patients in the experimental group. This means that it exerts its action at the respiratory-tract level without absorption, which could lead to the appearance of systemic effects or pharmacological interactions. This fact is further confirmed by the absence of electrocardiographic alterations, nor hepatic or renal toxicity at the end of treatment. It is important to bear in mind, as previously mentioned, that progression to more severe stages of infection could also be associated with alterations in these markers [25,26].

Ethanol inhalation has been previously used for acute respiratory distress syndrome and alcohol withdrawal, among others. However, ethyl alcohol had never been used to treat respiratory infections prior to the outbreak of the COVID-19 pandemic. To our knowledge, three other clinical trials with inhaled/nebulized ethanol for the treatment of SARS-CoV-2 have been conducted [33,34,35]. One of them showed that symptomatology and clinical status improved significantly in the ethanol group [33]. As in our work, differences were found in CRP but not in SpO_2_. Even so, it should be noted that the study population, scale, and method of administration are not comparable with those used in our trial. In the other trial with published results, ethanol was administered together with dimethyl sulfoxide as an intranasal inhalation spray in volunteer health-care workers. Results showed that the incidence rates of COVID-19 in the control and intervention groups were 0.07 and 0.008, respectively [35]. As previously, important methodological differences make it difficult to establish direct comparisons with our work.

In the matter of the limitations of the present study, together with the selection of the progression scale, the most notable one was the small sample size. It is necessary to consider that the clinical trial took place in a center adapted to accommodate elderly patients with mild SARS-CoV-2 infection. After the initiation of vaccination, the number of positives in this population declined considerably in our area, forcing the closure of the center. For this reason, it was not possible to recruit the required number of subjects, a very important barrier to achieving statistical significance.

The present clinical trial demonstrates that the use of inhaled ethanol is safe for the treatment of early-stage SARS-CoV-2 infection in elderly patients. In addition, its non-specific mechanism of action would open the door to the evaluation of its use in other infectious respiratory pathologies. This trial represents an achievement in the field of hospital pharmacy, which has once again demonstrated its capacity for action with the implementation of an independent clinical trial in the early stages of the SARS-CoV-2 pandemic. This was possible thanks to the knowledge of pharmacology, and the mastery of compounding and drug repositioning. Further studies are required to obtain conclusive data and to evaluate the long-term efficacy and safety of inhaled ethanol for SARS-CoV-2 infection.

## Figures and Tables

**Table 1:** Demographic characteristics and risk factors of the intention-to-treat population.

	Ethanol (N = 38)	Placebo (N = 37)	Total (N = 75)	*p* Value
Demographic Characteristics				
Woman, N (%)	23 (60.5)	28 (75.7)	51 (68.0)	0.5759
Age, mean (SD)	82.5 (8.3)	83.6 (8.2)	83.0 (8.2)	0.9834
Advanced age ^1^, N (%)	30 (78.9)	32 (86.5)	62 (82.7)	0.5437
Cancer, N (%)	5 (13.2)	1 (2.7)	6 (8.0)	0.1997
Cardiovascular disease, N (%)	11 (28.9)	7 (18.9)	18 (24.0)	0.4189
Hypertension, N (%)	27 (71.1)	25 (67.6)	52 (69.3)	0.8054
Diabetes mellitus, N (%)	13 (34.2)	7 (18.9)	20 (26.7)	0.1921
Obesity, N (%)	7 (18.4)	3 (8.1)	10 (13.3)	0.3091
Symptoms				
Patients with at least one symptom, N (%)	20 (52.6)	17 (45.9)	37 (49.3)	0.6466
Fever, N (%)	4 (10.5)	3 (8.3)	7 (9.5)	>0.9999
General discomfort, N (%)	14 (36.8)	12 (32.4)	26 (34.7)	0.8093
Dyspnea, N (%)	4 (10.5)	1 (2.7)	5 (6.7)	0.3580
Cough, N (%)	15 (39.5)	12 (32.4)	27 (36.0)	0.6321
Loss of smell, N (%)	1 (2.6)	0 (0.0)	1 (1.3)	>0.9999
Loss of taste, N (%)	0 (0.0)	0 (0.0)	0 (0.0)	-
Diarrhea, N (%)	1 (2.6)	0 (0.0)	1 (1.3)	>0.9999
Abdominal pain, N (%)	1 (2.6)	0 (0.0)	1 (1.3)	>0.9999
Days with symptoms until admission, mean (SD)	1.7 (1.5)	1.6 (1.4)	1.7 (1.5)	0.8006

^1^ Advanced age: ≥75 years old.

## Data Availability

Not applicable.

## Supplementary Materials

The following supporting information can be downloaded at: https://www.mdpi.com/article/10.3390/pharmaceutics15020667/s1, Table S1. Inclusion and exclusion criteria of participants in the clinical trial; Table S2. Modified WHO Clinical Progression Scale; Table S3. Classification of blood biomarkers as indicators of efficacy and/or safety; Table S4. Chronic treatments grouped by therapeutic groups according to the Anatomical Therapeutic Chemical (ATC) Classification System at the beginning of the study in the intention-to-treat population. Fisher’s exact test; Table S5. Classification of patients per visit according to the modified WHO Clinical Progression Scale; Table S6. Number of patients who progressed and/or died per visit with respect to the previous visit. Fisher’s exact test; Table S7. Oxygen saturation (%) throughout the study in the intention-to-treat population. U Mann–Whitney test; Table S8. Patients who presented hypoxia (defined as oxygen saturation <91%) throughout the study according to each visit in the intention-to-treat population. Fisher’s test; Table S9. Temperature (°C) throughout the study in the intention-to-treat population. U Mann–Whitney test; Table S10. Administration of antipyretics in the different study visits in the intention-to-treat population. Fisher’s test; Table S11. Analytical parameters collected at the baseline study visit and at the end-of-treatment visit (day 5) expressed as median and interquartile ranges. U Mann–Whitney test; Table S12. Percentage change in analytical parameters between baseline visit and end-of-treatment visit (day 5). U Mann–Whitney test; Table S13. Equivalent doses of prednisone/day and the total equivalent doses of prednisone administered in both groups.
