# Supplementary material for: Efficacy and Safety of Inhaled Ethanol in Early-Stage SARS-CoV-2 Infection in Older Adults: A Phase II Randomized Clinical Trial
Source: Pharmaceutics. 2023 Feb 16;15(2):667. doi: 10.3390/pharmaceutics15020667 (PMC9966500; doi:10.3390/pharmaceutics15020667)
Supplement: Supplementary file 1 [file pharmaceutics-15-00667-s001.zip › pharmaceutics-2152023-supplementary.pdf]

Supplementary Material: Efficacy and Safety of Inhaled Ethanol in Early-Stage SARS-CoV-2 Infection in Older Adults: A Phase II Randomized Clinical Trial

Ana Castro-Balado [1,2,3,†], Ignacio Novo-Veleiro [4,†], Néstor Vázquez-Agra [4,†], Gema Barbeito-Castiñeiras [5], Ana Estany-Gestal [6], Rocío Trastoy-Pena [5], Miguel González-Barcia [1,2], Irene Zarra-Ferro [1,2], María Carmen del Río-Garma [7], Carlos Crespo-Diz [8], Olga Delgado-Sánchez [9], Francisco J. Otero-Espinar [3], Cristina Mondelo-García [1,2,*], Antonio Pose-Reino [4,*] and Anxo Fernández-Ferreiro [1,2,*]

Table S1. Inclusion and exclusion criteria for of participants in the clinical trial.

**Inclusion criteria**
Institutionalized men or women aged 65 or older at the time of signing the informed consent.
Patients able to understand the trial procedures and agree to participate.
Diagnosis of COVID-19 confirmed by RT-PCR [1].
Initial stage of the disease diagnosed by:<br>- Less than 7 days from the appearance of the first symptoms.<br>- Absence of dyspnea<br>- Absence of pneumonia<br>- Oxygen saturation >93% or pO_2 >70 mmHg<br>- Respiratory rate <25 min^{-1}
Signature and date of the informed consent before any activity related to the study, including the evaluations necessary for the selection.
**Exclusion criteria**
Impaired renal function (creatinine > 2.5 times the normal limit), need for haemofiltration or impaired liver function (ALT or AST > 3 times the normal limits) or diagnosis of severe renal failure.
Hypersensitivity, allergy, or contraindications to study treatments.
Inability to administer oxygen therapy using Ventimask®.
Diagnosis of any other pathology that, in the investigator's opinion, may increase the risk of the subject or reduce the possibilities of obtaining satisfactory data to achieve the objectives of the study.
Consumption of any other drug that could incapacitate you, in the opinion of the investigator, to participate in the study.
Other circumstances or difficulties that, in the opinion of the investigator, may increase the risk of the subject or reduce the possibilities of obtaining satisfactory data to achieve the objectives of the study.
Participation in another clinical study where they have received an investigational drug in the 24 weeks prior to signing the informed consent.
Patients diagnosed with chronic broncho-pneumopathy.
Patients with a history of epilepsy.
Patients with a history of alcoholism.
Treatment with anticonvulsant drugs used to treat epilepsy and that have a higher degree of interaction with ethanol: topiramate, carbamazepine, perampanel and stiripentol

Treatment with drugs that, administered concomitantly with ethanol, can cause the so-called "disulfiram-like effect": disulfiram, metronidazole, tinidazole, chloramphenicol, levamisole, nitrofurantoin, isoniazid and griseofulvin.

[1] RT-PCR: reverse-transcriptase polymerase chain reaction; pO2: oxygen partial pressure; ALT: alanine aminotransferase; AST: aspartate aminotransferase.

Table S2. Modified WHO Clinical Progression Scale.

Patient State	Descriptor
**Uninfected**	Uninfected, no clinical or virologic signs of infection
**Ambulatory mild disease**	Outpatient, without limitation of activities
	Outpatient, without limitation of activities
**Hospitalized: moderate disease**	Hospitalized, without oxygen therapy
	Hospitalized with oxygen therapy by mask or nasal prongs
**Hospitalized: severe diseases**	Hospitalized with non-invasive mechanical ventilation or high-flow oxygen therapy
	Hospitalized with invasive mechanical ventilation
	Hospitalized with mechanical ventilation + ECMO [1] + organic support
**Dead**	Dead

[1] ECMO: extracorporeal membrane oxygenation.

Table S3. Classification of blood biomarkers as indicators of efficacy and/or safety.

	Efficacy	Safety
**Basic biochemistry**	alanine aminotransferase (ALT), aspartate aminotransferase (AST), alkaline phosphatase (AP), gamma glutamyl transferase (GGT), bilirubin, albumin, creatinine, and urea	liver safety: alanine aminotransferase (ALT), aspartate aminotransferase (AST), alkaline phosphatase (AP), gamma glutamyl transferase (GGT), bilirubin and albumin<br>renal safety: creatinine, and urea
**Complete blood count**	hemoglobin, platelets, leucocytes, lymphocytes neutrophils, monocytes, basophils, eosinophils	
**Coagulation parameters**	prothrombin time (PT), activated partial thromboplastin time (aPTT), d-dimer, fibrinogen	
**Inflammatory parameters**	C-reactive protein (CRP), procalcitonin (PCT), creatine kinase (CK), lactate	

dehydrogenase (LHD), troponin-I (TnI) and interleukin-6 (IL-6)

Table S4. Chronic treatments grouped by therapeutic groups according to the Anatomical Therapeutic Chemical (ATC) Classification System at the beginning of the study in the intention-to-treat population. Fisher's exact test.

Therapeutic group	Ethanol (N=38)	Placebo (N=37)	Overall (N=75)	p-value
	N (%)	N (%)	N (%)	
**Additives for IV solutions**	1 (2.6%)	0 (0.0%)	1 (1.3%)	>0.9999
**Adrenergic drugs for systemic use**	1 (2.6%)	0 (0.0%)	1 (1.3%)	>0.9999
**Inhaled Adrenergic agents**	4 (10.5%)	1 (2.7%)	5 (6.7%)	0.3580
**Potassium sparing agents**	4 (10.5%)	1 (2.7%)	5 (6.7%)	0.3580
**Anticholinergic agents**	2 (5.3%)	1 (2.7%)	3 (4.0%)	>0.9999
**Anti-inflammatory agents**	0 (0.0%)	1 (2.7%)	1 (1.3%)	0.4933
**Antiparathyroid agents**	1 (2.6%)	0 (0.0%)	1 (1.3%)	>0.9999
**Antithrombotic agents**	34 (89.5%)	31 (83.8%)	65 (86.7%)	0.5161
**Beta-blocking agents**	10 (26.3%)	6 (16.2%)	16 (21.3%)	0.2858
**Agents against peptic ulcer and gastroesophageal reflux (GER/GORD)**	35 (92.1%)	34 (91.9%)	69 (92.0%)	>0.9999
**Agents against functional disorders of the stomach**	1 (2.6%)	0 (0.0%)	1 (1.3%)	>0.9999
**Dopaminergic agents**	1 (2.6%)	0 (0.0%)	1 (1.3%)	>0.9999
**Lipid-modifying agents**	16 (42.1%)	8 (21.6%)	24 (32.0%)	0.0573
**Anxiolytics**	12 (31.6%)	10 (27.0%)	22 (29.3%)	0.6651
**Hormone antagonists and related agents**	0 (0.0%)	1 (2.7%)	1 (1.3%)	0.4933
**Angiotensin II receptor antagonists**	14 (36.8%)	12 (32.4%)	26 (34.7%)	0.6883
**Antiandrogens**	1 (2.6%)	0 (0.0%)	1 (1.3%)	>0.9999
**Class I and III antiarrhythmics**	0 (0.0%)	1 (2.7%)	1 (1.3%)	0.4933
**Beta-lactam antibiotics, penicillins**	0 (0.0%)	2 (5.4%)	2 (2.7%)	0.2400
**Antidepressants**	9 (23.7%)	19 (51.4%)	28 (37.3%)	0.0133
**Antiepileptics**	5 (13.2%)	9 (24.3%)	14 (18.7%)	0.2147
**Antifungals for topical use**	0 (0.0%)	1 (2.7%)	1 (1.3%)	0.4933
**Antipsychotics**	11 (28.9%)	13 (35.1%)	24 (32.0%)	0.5657
**Direct-acting antivirals**	0 (0.0%)	1 (2.7%)	1 (1.3%)	0.4933
**Belladonna and derivatives**	0 (0.0%)	1 (2.7%)	1 (1.3%)	0.4933
**Selective calcium channel blockers with direct cardiac effects**	0 (0.0%)	1 (2.7%)	1 (1.3%)	0.4933
**Selective calcium channel blockers with mainly vascular effects**	7 (18.4%)	10 (27.0%)	17 (22.7%)	0.3735
**Corticosteroids for systemic use**	16 (42.1%)	15 (40.5%)	31 (41.3%)	0.8906
**High ceiling diuretics**	12 (31.6%)	9 (24.3%)	21 (28.0%)	0.4842
**Low ceiling diuretics**	2 (5.3%)	3 (8.1%)	5 (6.7%)	0.6745

**Dopa and dopa derivatives**	1 (2.6%)	0 (0.0%)	1 (1.3%)	>0.9999
**Expectorants, excluding combinations with cough suppressants**	1 (2.6%)	0 (0.0%)	1 (1.3%)	>0.9999
**Anti-dementia drugs**	0 (0.0%)	4 (10.8%)	4 (5.3%)	0.0543
**Hypoglycemic drugs excluding insulins**	9 (23.7%)	5 (13.5%)	14 (18.7%)	0.2584
**Drugs for constipation**	9 (23.7%)	3 (8.1%)	12 (16.0%)	0.0658
**Drugs used in benign prostatic hypertrophy**	6 (15.8%)	0 (0.0%)	6 (8.0%)	0.0253
**Cardiac glycosides**	2 (5.3%)	4 (10.8%)	6 (8.0%)	0.4303
**Hypnotics and sedatives**	10 (26.3%)	9 (24.3%)	19 (25.3%)	0.8428
**ACE inhibitors**	7 (18.4%)	9 (24.3%)	16 (21.3%)	0.5327
**Insulins and analogues**	5 (13.2%)	3 (8.1%)	8 (10.7%)	0.7110
**Macrolides, lincosamides and streptogramins**	4 (10.5%)	3 (8.1%)	7 (9.3%)	>0.9999
**mydriatics and cycloplegics**	0 (0.0%)	1 (2.7%)	1 (1.3%)	0.4933
**Nutritional modules**	1 (2.6%)	0 (0.0%)	1 (1.3%)	>0.9999
**Opioids**	4 (10.5%)	5 (13.5%)	9 (12.0%)	0.7361
**Other inhaled agents for obstructive airway conditions**	8 (21.1%)	6 (16.2%)	14 (18.7%)	0.5910
**Other diagnostic agents**	0 (0.0%)	3 (8.1%)	3 (4.0%)	0.1151
**Other analgesics and antipyretics**	7 (18.4%)	7 (18.9%)	14 (18.7%)	0.9559
**Beta-lactam antibacterials**	8 (21.1%)	4 (10.8%)	12 (16.0%)	0.2264
**Other ophthalmological**	1 (2.6%)	1 (2.7%)	2 (2.7%)	>0.9999
**Other agents for the heart**	1 (2.6%)	0 (0.0%)	1 (1.3%)	>0.9999
**Other products for urological use, including antispasmodics**	1 (2.6%)	1 (2.7%)	2 (2.7%)	>0.9999
**Other mineral supplements**	1 (2.6%)	0 (0.0%)	1 (1.3%)	>0.9999
**Anti-gout agents**	2 (5.3%)	3 (8.1%)	5 (6.7%)	0.6745
**Antithyroid agents**	0 (0.0%)	1 (2.7%)	1 (1.3%)	0.4933
**Preparations with iron**	8 (21.1%)	4 (10.8%)	12 (16.0%)	0.2264
**Anti-glaucoma and miotic agents**	4 (10.5%)	3 (8.1%)	7 (9.3%)	>0.9999
**Agents against vertigo**	0 (0.0%)	4 (10.8%)	4 (5.3%)	0.0543
**Thyroid hormone agents**	5 (13.2%)	1 (2.7%)	6 (8.0%)	0.1997
**Nonsteroidal anti-inflammatory and antirheumatic products**	0 (0.0%)	1 (2.7%)	1 (1.3%)	0.4933
**Propellants**	1 (2.6%)	2 (5.4%)	3 (4.0%)	0.6148
**Psychostimulants, agents used for ADHD, and nootropics**	1 (2.6%)	1 (2.7%)	2 (2.7%)	>0.9999
**Antibacterial quinolones**	2 (5.3%)	3 (8.1%)	5 (6.7%)	0.6745
**IV Solutions**	1 (2.6%)	0 (0.0%)	1 (1.3%)	>0.9999
**Mineral supplements**	4 (10.5%)	3 (8.1%)	7 (9.3%)	>0.9999
**Cough suppressants, excluding combinations with expectorants**	3 (7.9%)	3 (8.1%)	6 (8.0%)	>0.9999
**Peripheral vasodilators**	3 (7.9%)	1 (2.7%)	4 (5.3%)	0.6148
**Vasodilators used in heart disease**	2 (5.3%)	1 (2.7%)	3 (4.0%)	>0.9999

**Vitamin B1, alone and in combination with vitamins B6 and B12**	1 (2.6%)	0 (0.0%)	1 (1.3%)	>0.9999
**Vitamin B12 and folic acid**	1 (2.6%)	2 (5.4%)	3 (4.0%)	0.6148

Table S5. Classification of patients per visit according to the modified WHO Clinical Progression Scale.

	Ethanol (N=38)		Placebo (N=37)		Total (N=75)	
**Modified WHO Clinical Progression Scale**	**N**	**%**	**N**	**%**	**N**	**%**
**Baseline**						
**Not infected, no clinical or virological signs of infection.**	0	0.0	0	0.0	0	0.0
**Ambulatory, without limitation of activities**	32	84.2	32	86.5	64	85.3
**Ambulatory with activity limitation**	6	15.8	5	13.5	11	14.7
**Hospitalized, without oxygen therapy**	0	0.0	0	0.0	0	0.0
**Hospitalized with oxygen therapy by mask or nasal goggles**	0	0.0	0	0.0	0	0.0
**Hospitalized with noninvasive mechanical ventilation or high-flow oxygen therapy**	0	0.0	0	0.0	0	0.0
**Hospitalized with invasive mechanical ventilation**	0	0.0	0	0.0	0	0.0
**Hospitalized with mechanical ventilation + ECMO + organ support**	0	0.0	0	0.0	0	0.0
**Death**	0	0.0	0	0.0	0	0.0
**Day 5**						
**Not infected, no clinical or virological signs of infection.**	0	0.0	0	0.0	0	0.0
**Ambulatory, without limitation of activities**	32	84.2	29	78.4	61	81.3
**Ambulatory with activity limitation**	1	2.6	3	8.1	4	5.3
**Hospitalized, without oxygen therapy**	0	0.0	1	2.7	1	1.3
**Hospitalized with oxygen therapy by mask or nasal goggles**	5	13.2	4	10.8	9	12.0
**Hospitalized with noninvasive mechanical ventilation or high-flow oxygen therapy**	0	0.0	0	0.0	0	0.0
**Hospitalized with invasive mechanical ventilation**	0	0.0	0	0.0	0	0.0
**Hospitalized with mechanical ventilation + ECMO + organ support**	0	0.0	0	0.0	0	0.0
**Death**	0	0.0	0	0.0	0	0.0

**Day 14**						
**Not infected, no clinical or virological signs of infection.**	12	31.6	15	40.5	27	36.0
**Ambulatory, without limitation of activities**	18	47.4	15	40.5	33	44.0
**Ambulatory with activity limitation**	0	0.0	3	8.1	3	4.0
**Hospitalized, without oxygen therapy**	3	7.9	0	0.0	3	4.0
**Hospitalized with oxygen therapy by mask or nasal goggles**	2	5.3	4	10.8	6	8.0
**Hospitalized with noninvasive mechanical ventilation or high-flow oxygen therapy**	1	2.6	0	0.0	1	1.3
**Hospitalized with invasive mechanical ventilation**	0	0.0	0	0.0	0	0.0
**Hospitalized with mechanical ventilation + ECMO + organ support**	0	0.0	0	0.0	0	0.0
**Death**	2	5.3	0	0.0	2	2.7
**Day 28**						
**Not infected, no clinical or virological signs of infection.**	35	94.6	31	83.8	66	89.2
**Ambulatory, without limitation of activities**	0	0.0	3	8.1	3	4.1
**Ambulatory with activity limitation**	0	0.0	0	0.0	0	0.0
**Hospitalized, without oxygen therapy**	0	0.0	0	0.0	0	0.0
**Hospitalized with oxygen therapy by mask or nasal goggles**	1	2.7	1	2.7	2	2.7
**Hospitalized with noninvasive mechanical ventilation or high-flow oxygen therapy**	0	0.0	0	0.0	0	0.0
**Hospitalized with invasive mechanical ventilation**	0	0.0	0	0.0	0	0.0
**Hospitalized with mechanical ventilation + ECMO + organ support**	0	0.0	0	0.0	0	0.0
**Death**	2	5.3	2	5.4	4	5.3

Table S6. Number of patients who progressed and/or died per visit with respect to the previous visit. Fisher's exact test.

Visit	Ethanol (N=38)	Placebo (N=37)	p
**Day 5, N (%)**	5 (13.2)	6 (16.2)	0.7543
**Day 14, N (%)**	8 (21.1)	8 (21.6)	>0.9999
**Day 28, N (%)**	8 (21.1)	8 (21.6)	>0.9999

Table S7. Oxygen saturation (%) throughout the study in the intention-to-treat population. U Mann–Whitney test.

Visit	Group	Median	IQR	Range	p
**Baseline**	**Ethanol (N=38)**	95.0	95.0-96.0	91.0-97.0	0.4482
	**Placebo (N=37)**	96.0	95.0-96.0	91.0-98.0	
**Day 5**	**Ethanol (N=38)**	95.5	95.0-96.0	86.0-96.0	0.7318
	**Placebo (N=37)**	96.0	95.0-96.0	85.0-98.0	
**Day 10**	**Ethanol (N=37)**	96.0	95.0-96.0	90.0-96.0	0.5433
	**Placebo (N=37)**	95.0	95.0-96.0	88.0-97.0	
**Day 14**	**Ethanol (N=37)**	96.0	95.0-96.0	88.0-98.0	0.4300
	**Placebo (N=37)**	95.0	95.0-96.0	85.0-97.0	
**Day 21**	**Ethanol (N=36)**	96.0	95.0-96.0	92.0-97.0	0.4182
	**Placebo (N=35)**	96.0	95.0-96.0	91.0-97.0	
**Day 28**	**Ethanol (N=36)**	96.0	95.0-96.0	93.0-97.0	0.8489
	**Placebo (N=35)**	96.0	95.0-96.0	89.0-96.0	

Table S8. Patients who presented hypoxia (defined as oxygen saturation <91%) throughout the study according to each visit in the intention-to-treat population. Fisher's test.

Visit	Ethanol (N=38)	Placebo (N=37)	p
**Basal, N (%)**	0 (0)	0 (0)	-
**Day 5, N (%)**	2 (5.3)	2 (5.4)	>0.9999
**Day 10, N (%)**	2 (5.4)	1 (2.7)	>0.9999
**Day 14, N (%)**	1 (2.7)	3 (8.1)	0.6145
**Day 21, N (%)**	0 (0)	0 (0)	-
**Day 28, N (%)**	0 (0)	1 (2.9)	0.4930

Table S9. Temperature (°C) throughout the study in the intention-to-treat population. U Mann–Whitney test.

Visit	Group	Median	IQR	Range	p
**Baseline**	**Ethanol (N=38)**	36.2	36.1-36.4	35.7-37.6	0.2894
	**Placebo (N=37)**	36.3	36.2-36.5	35.7-38.0	
**Day 2**	**Ethanol (N=38)**	36.2	36.0-36.3	35.0-38.0	0.1125
	**Placebo (N=36)**	36.2	36.1-36.4	35.6-38.0	
**Day 3**	**Ethanol (N=38)**	36.2	36.0-36.3	35.7-38.5	0.6251
	**Placebo (N=35)**	36.2	36.0-36.3	35.7-37.0	
**Day 5**	**Ethanol (N=38)**	36.2	36.0-36.4	35.5-38.1	0.5482
	**Placebo (N=37)**	36.2	36.0-36.3	35.7-38.0	

Table S10. Administration of antipyretics in the different study visits in the intention-to-treat population. Fisher's test.

Visit	Ethanol (N=38)	Placebo (N=37)	p
**Baseline, N (%)**	7 (18.4)	4 (10.8)	0.5161
**Day 2, N (%)**	5 (13.2)	5 (13.5)	>0.9999
**Day 3, N (%)**	4 (10.5)	5 (13.5)	0.7361
**Day 5, N (%)**	6 (15.8)	6 (16.2)	>0.9999

Table S11. Analytical parameters collected at the baseline study visit and at the end-of-treatment visit (day 5) expressed as median and interquartile ranges. U Mann–Whitney test.

Parameter	Visit	Ethanol	Placebo	p
**Basic biochemistry**				
**Glucose (mg/dL)**	**Baseline**	90.5 (80.0-112.0)	88.0 (79.5-107.0)	0.7653
	**Day 5**	96.0 (79.0-111.0)	85.5 (76.0-97.5)	0.1815
**Urea (mg/dl)**	**Baseline**	54.5 (38.5-80.0)	41.0 (39.0-61.5)	0.1572
	**Day 5**	59.0 (43.0-82.0)	48.5 (35.5-73.0)	0.1564
**Creatinine (mg/dl)**	**Baseline**	1.0 (0.7-1.1)	0.8 (0.7-1.0)	0.2437
	**Day 5**	0.9 (0.7-1.1)	0.8 (0.7-0.8)	0.1676
**Bilirubin (mg/dl)**	**Baseline**	0.5 (0.3-0.6)	0.5 (0.4-0.6)	0.8731
	**Day 5**	0.4 (0.4-0.6)	0.4 (0.3-0.6)	0.9034
**GOT (AST) (U/I)**	**Baseline**	23.0 (18.5-30.5)	23.0 (20.0-29.0)	0.6742
	**Day 5**	24.0 (21.0-29.0)	29.5 (20.0-47.0)	0.1380
**GPT (ALT) (U/I)**	**Baseline**	20.5 (13.5-30.0)	18.0 (14.0-30.0)	0.8790
	**Day 5**	24.0 (17.0-31.0)	30.0 (17.0-53.5)	0.3573
**Gamma GT (GGT) (U/I)**	**Baseline**	22.5 (14.0-38.0)	25.0 (16.0-44.5)	0.4777
	**Day 5**	27.5 (14.0-41.0)	27.5 (18.0-69.0)	0.4392
**Albumin (g/dl)**	**Baseline**	3.8 (3.6-4.0)	3.9 (3.6-4.0)	0.2869
	**Day 5**	3.8 (3.6-4.0)	3.8 (3.5-3.9)	0.6554
**Calcium (mEq/l)**	**Baseline**	8.8 (8.4-9.0)	8.7 (8.5-9.1)	0.5799
	**Day 5**	8.8 (8.4-9.0)	8.7 (8.4-9.1)	0.7862
**Inorganic phosphate (mEq/l)**	**Baseline**	3.2 (2.8-3.8)	3.3 (3.0-3.5)	0.6041
	**Day 5**	3.1 (2.9-3.4)	3.2 (2.8-3.5)	0.6041
**Complete blood count**				
**Hemoglobin (g/dl)**	**Baseline**	12.6 (11.6-13.6)	12.9 (12.4-13.7)	0.1259
	**Day 5**	12.6 (11.4-13.5)	12.8 (11.9-13.9)	0.4121
**Platelets (x10^3/µL)**	**Baseline**	190.5 (152.5-229.5)	178.0 (144.0-217.0)	0.2384
	**Day 5**	218.5 (173.0-273.0)	186.5 (153.5-249.5)	0.1679
**Leucocytes (x10^3/µL)**	**Baseline**	4.5 (3.4-6.5)	4.8 (3.7-6.1)	0.9551
	**Day 5**	5.7 (4.5-7.1)	5.0 (4.3-7.1)	0.4019
**Lymphocytes (x10^3/µL)**	**Baseline**	1.2 (0.8-1.7)	1.5 (1.0-2.0)	0.3384

	**Day 5**	1.3 (0.9-1.8)	1.3 (0.9-1.8)	0.7333
**Neutrophils (x10³/μL)**	**Baseline**	2.6 (1.9-3.3)	2.5 (2.0-3.3)	0.7227
	**Day 5**	3.4 (2.5-5.1)	3.0 (2.2-4.6)	0.2494
**Monocytes (x10³/μL)**	**Baseline**	0.430 (0.330-0.490)	0.420 (0.310-0.580)	0.7226
	**Day 5**	0.430 (0.350-0.540)	0.420 (0.320-0.525)	0.5960
**Basophils (x10³/μL)**	**Baseline**	0.020 (0.010-0.030)	0.020 (0.010-0.020)	0.5920
	**Day 5**	0.020 (0.010-0.030)	0.020 (0.010-0.030)	0.8839
**Eosinophils (x10³/μL)**	**Baseline**	0.04 (0.01-0.12)	0.03 (0.01-0.11)	0.8960
	**Day 5**	0.06 (0.02-0.17)	0.04 (0.01-0.12)	0.3541
**Coagulation parameters**				
**Prothrombin time (s)**	**Baseline**	11.9 (11.6-12.5)	12.1 (11.6-12.6)	0.9706
	**Day 5**	12.2 (11.6-12.9)	12.1 (11.6-12.9)	0.9678
**aPTT (s)**	**Baseline**	29.3 (27.3-32.9)	30.0 (28.3-32.5)	0.2667
	**Day 5**	29.3 (25.8-30.6)	28.5 (27.6-31.3)	0.3746
**Fibrinogen (mg/dL)**	**Baseline**	388.0 (324.0-449.0)	384.5 (319.0-446.0)	0.8291
	**Day 5**	410.0 (368.0-494.0)	483.0 (412.0-555.0)	0.0939
**D-dimer (ng/mL)**	**Baseline**	714.0 (437.0-1126.0)	559.0 (410.0-918.0)	0.3384
	**Day 5**	575.0 (433.0-1212.0)	669.0 (460.0-1159.0)	0.9214
**Inflammatory parameters**				
**Troponin-I (ng/mL)**	**Baseline**	0.04 (0.02-0.06)	0.03 (0.00-0.04)	0.2360
	**Day 5**	0.02 (0.00-0.03)	0.02 (0.00-0.03)	0.5476
**Creatinine Kinase (U/L)**	**Baseline**	70.0 (48.0-104.0)	59.5 (33.0-110.5)	0.2057
	**Day 5**	40.5 (27.0-60.0)	48.0 (34.0-85.0)	0.3310
**Lactate Dehydrogenase (UI/I)**	**Baseline**	328.0 (297.0-386.0)	352.0 (294.0-420.0)	0.4270
	**Day 5**	348.0 (299.0-401.0)	389.0 (309.5-432.5)	0.2584
**C-reactive protein (mg/dL)**	**Baseline**	0.81 (0.22-1.48)	0.73 (0.23-1.67)	0.9820
	**Day 5**	0.46 (0.14-1.48)	1.11 (0.41-3.02)	0.0529
**Procalcitonin (ng/mL)**	**Baseline**	0.06 (0.05-0.11)	0.05 (0.04-0.08)	0.0306
	**Day 5**	0.06 (0.04-0.08)	0.05 (0.04-0.08)	0.8825
**IL-6 (pg/mL)**	**Baseline**	0.9 (0.6-1.3)	1.1 (0.8-1.9)	0.0990
	**Day 5**	0.8 (0.5-1.4)	1.2 (0.7-1.9)	0.0540

Table S12. Percentage change in analytical parameters between baseline visit and end-of-treatment visit (day 5). U Mann–Whitney test.

Parameter	Ethanol (% median)	Placebo (% median)	p
**Basic biochemistry**			
**Glucose (mg/dL)**	1.98	0.00	0.6008
**Urea (mg/dl)**	1.22	6.06	0.4900
**Creatinine (mg/dl)**	-2.97	-3.57	0.9345
**Bilirubin (mg/dl)**	0.00	0.00	0.8606
**GOT (AST) (U/I)**	0.00	4.86	0.5028
**GPT (ALT) (U/I)**	15.00	9.09	0.6999
**Gamma GT (GGT) (U/I)**	8.63	6.67	0.6086
**Albumin (g/dl)**	0.00	-2.50	0.0408
**Calcium (mEq/l)**	0.00	0.00	0.5684
**Inorganic phosphate (mEq/l)**	-3.33	-3.97	0.8051
**Complete blood count**			
**Hemoglobin (g/dl)**	0.00	0.00	0.1327
**Platelets (x10^3/μL)**	11.13	8.67	0.7377
**Leucocytes (x10^3/μL)**	14.41	0.00	0.1364
**Lymphocytes (x10^3/μL)**	-1.96	0.00	0.7561
**Neutrophils (x10^3/μL)**	22.08	4.70	0.1425
**Monocytes (x10 μ^3/L)**	3.08	0.00	0.6331
**Basophils (x10^3/μL)**	0.00	0.00	0.7962
**Eosinophils (x10^3/μL)**	0.00	0.00	0.3131
**Coagulation parameters**			
**Prothrombin time (s)**	0.84	0.74	0.9678
**aPTT (s)**	-2.39	-4.41	0.8193
**Fibrinogen (mg/dL)**	13.58	15.42	0.3361
**D-dimer (ng/mL)**	-8.66	0.00	0.2432
**Inflammatory parameters**			
**Troponin-I (ng/mL)**	-38.54	-1.28	0.0772
**Creatinine Kinase (U/L)**	-36.08	-11.65	0.0020
**Lactate Dehydrogenase (UI/I)**	1.56	5.59	0.3083
**C-reactive protein (mg/dL)**	0.66	50.56	0.0379
**Procalcitonin (ng/mL)**	-14.29	0.00	0.1257
**IL-6 (pg/mL)**	0.00	0.00	0.6411

Table S13. Equivalent doses of prednisone/day and the total equivalent doses of prednisone administered in both groups.

	Daily prednisone dose prescribed			Total prednisone administered			
	**Median**	**IQR**	**p**	**Mean**	**SD**	**p**	**n**
**Ethanol**	48.1	39.9-56.2	0.4912	919.2	752.9	0.2104	19
**Placebo**	51.7	37.0-66.4		657.8	481.6		19
